# miR-128 plays a critical role in murine osteoclastogenesis and estrogen deficiency-induced bone loss

**DOI:** 10.7150/thno.42982

**Published:** 2020-03-04

**Authors:** Gengyang Shen, Hui Ren, Qi Shang, Zhida Zhang, Wenhua Zhao, Xiang Yu, Jingjing Tang, Zhidong Yang, De Liang, Xiaobing Jiang

**Affiliations:** 1 The First Affiliated Hospital of Guangzhou University of Chinese Medicine, Guangzhou 510405, China; 2 Guangzhou University of Chinese Medicine, Guangzhou 510405, China; 3 Lingnan Medical Research Center of Guangzhou University of Chinese Medicine, Guangzhou 510405, China

**Keywords:** miR-128, osteoclastogenesis, PMOP, ovariectomy, aging, inflammation

## Abstract

Postmenopausal osteoporosis (PMOP) is a severe health issue faced by postmenopausal women. microRNA-128 (miR-128) is associated with aging, inflammatory signaling, and inflammatory diseases, such as PMOP. It has also been reported to modulate *in vitro* osteogenic/adipogenic differentiation. However, its function in osteoclast formation is unknown.

**Methods**: First, the expression of *miR-128* and nuclear factor of activated T cells 1 (*Nfatc1,* bone resorption master marker) was investigated in bone tissues derived from PMOP patients, while their correlation to each other was also investigated. The levels of *miR-128* and *Nfatc1* in bone specimens and bone marrow-derived macrophages (BMMs) from mice subjected to ovariectomy (OVX) were also assayed. Next, we employed mice BMMs modified for overexpression and inhibition of miR-128 levels to determine its effect on osteoclast differentiation. Moreover, we generated osteoclastic miR-128 conditional knockout (*miR-128^Oc-/-^*) mice and isolated miR-128 deletion-BMMs to observe its biological function on bone phenotype and osteoclastogenesis *in vivo*, respectively. The *miR-128^Oc-/-^* BMMs were used to explore the downstream regulatory mechanisms using pull-down, luciferase reporter, and western-blotting assays. Finally, the impact of miR-128 deficiency on OVX-induced bone loss in mice was evaluated.

**Results**: The miR-128 level was found to be positively correlated with the increase in *Nfatc1* level in mouse/human bone specimens and mouse primary BMMs. *In vitro* experiments demonstrated miR-128 levels that were dependent on activity of osteoclast differentiation and miR-128 overexpression or inhibition in BMMs significantly increased or decreased osteoclastogenesis, respectively. *In vivo*, we revealed that osteoclastic miR-128 deletion remarkedly increased bone mass through the inhibition of osteoclastogenesis. Mechanistically, we identified sirtuin 1 (SIRT1) as the direct target of miR-128 at the post-transcriptional level during osteoclast differentiation. Increased levels of SIRT1 reduced nuclear factor κB (NF-κB) activity by decreasing the level of acetylation of Lysine 310, as well as inhibiting tumor necrosis factor-α (Tnf-α) and interleukin 1 (IL-1) expressions. Lastly, osteoclastic deletion of miR-128 significantly suppressed OVX-triggered osteoclastogenesis and exerted a protective effect against bone loss in mice.

**Conclusions**: Our findings reveal a critical mechanism for osteoclastogenesis that is mediated by the miR-128/SIRT1/NF-κB signaling axis, highlighting a possible avenue for the further exploration of diagnostic and therapeutic target molecules in PMOP.

## Introduction

Postmenopausal osteoporosis (PMOP) is a frequently encountered bone disorder that is responsible for an increased risk of disability of millions of individuals worldwide, as well as being a major socioeconomic burden 1. This disease is closely related with aging and inflammation resulting from estrogen deficiency and is mainly characterized by osteoclast differentiation and bone resorption 2, 3. Osteoclasts originate from macrophage/monocyte precursor cells. Macrophage colony-stimulating factor (M-CSF) and receptor activator of nuclear factor κB (NF-κB) ligand (RANKL) are two crucial molecules that are responsible for the formation of functional osteoclasts 4-7. Osteoclastogenesis occurs through stimulation of RANKL via the RANK receptor 8. This interaction results in the production of TNF receptor-associated factor 6 (TRAF6), which activates NF-κB and other signaling pathways that in turn enhances the transcriptional regulation of nuclear factor of activated T cells c1 (Nfatc1, the master transcription factor) 9, 10. Therefore, a more in depth understanding of aberrant osteoclastogenesis is essential for improvement of methods of diagnosis, treatment and prevention of PMOP.

microRNAs (miRNAs) are 22 nucleotides long small noncoding RNAs that are highly conserved. Their extensive and multifunctional roles in several physiological processes have been well documented 11, one of which is osteoclastogenesis 12-14. The complex process of osteoclastic bone resorption involves an alteration of the expression of several miRNAs, which in turn negatively regulate their target genes, leading to an impact on bone phenotypes 13, 14. Previously, Xia and colleagues reported that decreased miR-128 levels significantly suppressed the inflammatory response of rheumatoid arthritis (RA) and alleviates its progression by inhibiting the activity of the NF-κB pathway, a phenomenon that is mediated by tumor necrosis factor-α-induced protein 3 (TNFAIP3) 15. A subsequent study confirmed that miR-128 mediates tumor necrosis factor-α (Tnf-α)-induced inflammatory responses by targeting sirtuin 1 (SIRT1) in bone mesenchymal stem cells (BMSCs) 16. The expression of miR-128 was found to be significantly decreased in oxidized low-density lipoprotein (ox-LDL)-incubated macrophage cells RAW264.7, which are enriched for osteoclast progenitors 17. Other studies have also reported that the upregulation of miR-128 in inflamed chondrocytes represses chondrocyte autophagy and exacerbates knee osteoarthritis (OA) by disrupting Atg12 18. Further, Zhao et al. revealed that the expression level of miR-128 in bone samples of PMOP patients was significantly higher than that of non-PMOP patients 19. Although these findings suggest a potential interaction between miR-128 and osteoclastogenesis, not much has been found about the role of miR-128 in physiological and pathological bone metabolism, the latter of which is a predominant feature of osteoporosis.

Our investigations indicated that miR-128 is an important regulating factor of osteoclastogenesis in physiological conditions and bone metabolism- associated disease conditions, suggesting that the miR-128/SIRT1/NF-κB signaling axis may be responsible for osteoclastogenesis regulation. miR-128 directly targets SIRT1 by binding to its 3' UTR end during osteoclast differentiation. The increased SIRT1 levels inactivated NF-κB signaling by reducing acetylation of Lysine 310 [Acetyl-NF-κB p65 (Lys310)] and eventually suppressing osteoclastogenesis. Thus, targeting miR-128 expression of osteoclasts may represent an attractive therapeutic approach for PMOP prevention and treatment.

## Materials and Methods

### Human bone tissue preparation

The Ethics Committee of the First Affiliated Hospital of Guangzhou University of Chinese Medicine (No. ZYYECK [2016]028) approved all clinical experiments. This study enrolled PMOP patients (n=10) and non-PMOP patients (n=10) who had undergone spine-related surgeries. We obtained written informed consent from all patients. Inclusion criteria were patients with acute fragile lumbar fractures two weeks prior to selection and a clear indication of vertebroplasty or internal fixation. Those who were diabetic, who had malignancies or other systemic diseases diagnosed in the preceding 5 years were excluded from our cohort. Bone samples from the vertebrae were collected as previously described 20.

### Mice

The Laboratory Animal Center of Guangzhou University of Chinese Medicine supplied the wild Type (WT) C57BL/6 (B6) mice used in this study. Animal handling protocols were pre-approved by the Ethics Committee. 8-week-old female B6 mice (n = 8/group) were subjected to surgical OVX in order to establish a murine ovariectomy (OVX)-induced osteoporosis model. In brief, the mice were first weighed and anaesthetized using an intraperitoneal injection of Ketavet 25% (100 mg/mL) and xylazine 25% (20 mg/mL) in 60μL of phosphate-buffered saline (PBS). Osteoporosis was induced by carrying out bilateral ovariectomy using the external oblique muscle dissection approach. The control mice were subjected to laparotomy without OVX. All animals were reared under the same living environment with the same temperature, light, food and environmental conditions. Four weeks after the post-procedure, uterine atrophy was determined prior to vertebral dissection and analysis, which were conducted using micro-computed tomography (micro-CT) and bone histomorphometry methods. Mice with osteoclastic miR-128 conditionally knockout (hereafter referred to as *miR-128^Oc-/-^*) were created by breeding *miR-128^flox/flox^* mice (B6 background) with a *lysozyme M (LysM)-Cre* (B6 background) mice, which are known to express Cre under the influence of a LysM promoter. Controls for all *in vivo* experiments comprised of mice siblings that possessed a *miR-128^+/+^_LysM-Cre* genotype (designated as *WT* in this manuscript). All animal experiments were approved by the Ethics Committee of the First Affiliated Hospital of Guangzhou University of Chinese Medicine (No. TCMF1-2019030).

### Cell culture and transfection

For the osteoclastogenesis assays, bone marrow cells were cultured with M-CSF (100 ng/mL) alone for two days to recruit macrophages and then RANKL (50 ng/mL) was added to induce osteoclasts differentiation as previously described 21. Bone marrow cells were isolated using warm, serum-free Minimum Essential Medium Eagle Alpha Modifications (α-MEM) to flush out long bones. The resultant substrate was then centrifuged, and the cell suspension was plated and exposed to α-MEM supplemented with 10% fetal bovine serum (FBS) (Thermo Fisher, Waltham, Massachusetts, USA), 2 mM glutamine, as well as 100 mg/mL of both streptomycin and penicillin (Lonza, Basel, Switzerland). 5 days later, we then carried out TRAP staining, with TRAP-positive cells having more than three nuclei determined to be osteoclasts.

For the osteoblastogenesis assays, whole bone marrow cells were isolated from the long bones (femurs and tibias) of mice. The cells were cultured in α-MEM containing 50 μg/ml ascorbic acid (Sigma- Aldrich) and 10mM β-glycerophosphate (Sigma- Aldrich). The medium was changed every three days. After osteogenic induction for 7 days, the cells were fixed with 4% paraformaldehyde (Aladdin, Shanghai, China) for 15 min at room temperature, and a BCIP/ NBT Alkaline Phosphatase (ALP) Color Development Kit (Beyotime, Shanghai, China) was used for ALP staining. ALP activity was quantified using a commercial kit according to the manufacturer's protocol (Beyotime, Shanghai, China). After osteogenic induction for 14 days, the cells were fixed with 4% formaldehyde, followed by incubation with alizarin red staining (ARS) (Cyagen Biosciences, Guangzhou, China) to evaluate mineralized deposition formation. The calcium concentration was quantified using a standard calcium curve at 562 nm absorbance.

For the suppression or augmentation of miRNA, miRNA inhibitors or mimics were utilized (RiboBio, Guangzhou, China). Mouse BMMs were transfected with 100 nM of either the miR-128 mimic/miR-128 inhibitor or control mimic/control inhibitor using the Lipofectamine 2000 reagent. The effect of miR-128 overexpression or inhibition was identified using qRT-PCR. For gene knockdown, mouse SIRT1 and control small interfere RNAs (siRNAs) were procured from RiboBio (Guangzhou, China). There were three to five target-specific 19-25-nucleotide siRNAs in each siRNA used to knockdown the expression of target genes. Lipofectamine RNAimax (Invitrogen) was used to transfect mouse BMMs following the manufacturer's instruction. The effect of SIRT1 knockdown was identified by qRT-PCR and western blotting assays.

### Proliferation and apoptosis assay

The cell proliferation reagent, WST-1 (Sigma), was used to detect the proliferation of the BMMs 21. Each well contained 10 μl of reagent, except for three wells that only contained media (used for subtracting background reactions). After an hour of incubation at 37 °C, a microplate reader was used to interpret its absorbance at 450 nm. The cell death detection kit (Sigma) was utilized to analyze rate of BMM apoptosis in accordance with the manufacturer's protocols.

### RNA isolation and qRT-PCR

Bone tissues and BMMs were processed for RNA extraction. Reverse transcription of 1 μg of total RNA was conducted with a cDNA Synthesis kit (Takara) using 20μl reaction in order to produce cDNA. 20μl of SYBR Green qPCR SuperMix (Takara) with a real-time PCR machine (Bio-Rad) was used to analyze genes involved in perilacunar/canalicular remodeling, which included *Nfatc1*, *Traf6*, *Ctsk*, *c-Fos*, *c-Src, Tnf-α*, *IL-1, Runx2, Sp7, Alp* and* Ocn*. The primers (Supplementary Table 1) were designed by us and synthesized by Sangon Biotech (Shanghai, China). The cycling parameters used were 95℃ for 30 s followed by 40 cycles of 95℃ for 5 s and 60℃ for 30 s. The levels of gene expression were calculated using the 2^-ΔΔCt^ method.

For quantification of miRNA, total RNA was isolated, and the small RNA fraction was enriched using the mirVana miRNA Isolation Kit (Thermo Fisher Scientific) in compliance to the manufacturer's instructions. These small RNAs were then processed using TaqMan miRNA Reverse Transcription Kit (Thermo Fisher Scientific) in order to produce cDNA for quantitative RT-PCR analysis. TaqMan miRNA assays were used according to the manufacturer's recommendations (Thermo Fisher Scientific) to conduct real-time PCR assays. Data were normalized to levels of small nucleolar RNA (snRNA) U6.

### Western blotting analysis

Western Blotting analysis was performed as previously described 22. A RIPA lysis buffer (Beyotime) was used to lyse the cells for protein isolation, which was conducted using sodium dodecyl sulfate polyacrylamide gel electrophoresis (15%). The separated proteins were immunoblotted onto polyvinylidene fluoride membranes (Millipore, Shanghai, China). 5% non-fat milk was used to block endogenous reactions for 2 hours at room temperature. Further incubation for 24 h at 4℃ was done with primary antibodies against β-actin (1:3000; mouse; Cell Signalling Technology, Danvers, MA, USA), SIRT1 (1:500; mouse; Cell Signalling Technology), and Acetyl-NF-κB p65 (Lys310) (1:500; mouse; Cell Signalling Technology), and NF-κB p65 (1:500; human/mouse; Cell Signalling Technology). After washing three times for 10 min each with PBST, the membranes were incubated with the corresponding secondary antibodies for another 2 hours at room temperature. The final product was again rinsed thrice with PBST. Protein levels were determined by enhanced chemiluminescence (Bio- Rad Laboratories, Hercules, CA, USA) following the manufacturer's protocols. Band intensities were quantified using Image J software.

### Pull-down assay

Previous studies have documented the pull-down assay method mentioned below 22, 23. In brief, a DNA probe, prelabelled with biotin at the 3' terminal and was complementary to *SIRT1* mRNA, was constructed for *SIRT1* mRNA pull-down. Negative control comprised of a scrambled biotinylated probe (Thermo Fisher Scientific). Streptavidin-coated magnetic beads (Invitrogen) were used to incubate the probe at 25 ℃ for 1 h to generate probe-coated magnetic beads. The BMMs were harvested in a lysis buffer and the lysate was incubated with probe-coated magnetic beads at 37 ℃ for 3 h with constant rotation. After incubation, two washes with lysis buffer were performed and RNA was extracted using TRIzol regent (Invitrogen, CA, USA). The sequence of the SIRT1 probe is listed in Supplementary Table 1. The extracted RNA were analyzed using qRT-PCR.

### Luciferase assay

Genescript (Thermo Fisher Scientific) supplied all luciferase vectors used in this study. We constructed pMIR-report luciferase vectors containing binding sites for miR-128 on SIRT1's 3'-UTR to be used in the miRNA binding site tests. Binding specificity was tested using a mutant plasmid. The miR-128 binding site was mutated from CACUGUG to ACTGAGA. For the miR-128 promoter assay, miR-128 promoter regions containing SIRT1 binding sites were inserted into pGL3 basic reporter vectors (Promega, USA). Luciferase vectors and small RNA oligos were then transfected into the BMMs. A luciferase assay kit (Promega, USA) was used to test luciferase activity.

### Micro-CT

Mice L4 vertebrae were harvested and subjected to 48 hours of fixation in 4% paraformaldehyde before being analyzed by a high-resolution micro-CT imaging system (Skyscan, Kontich, Belgium). Scanner parameters were set as follows: 12 mm resolution, 100 μA current and 80 kV voltage. The L4 vertebrae trabecular bone was then analyzed using 3D model visualization software (mCTVol v2.0), data analysis software (CTAn v1.9) and volume reconstruction software (NRecon v1.6). Bone mineral density (BMD, mg/cm^2^), Trabecular thickness (Tb.Th, mm), trabecular separation (Tb.Sp, mm), trabecular number (Tb.N, /mm), connectivity density (Conn-Dens., mm) and trabecular bone volume per tissue volume (BV/TV, %) within a limited volume of interest (VOI) were measured.

### Bone histomorphometry analysis

Bone histomorphometry analysis was performed on the L3-L5 vertebrae, as previously described 25, 26. Femur sections were processed in Periodate- Lysine-Paraformaldehyde Fixative (TIANDZ) for 24h. A Shandon Finesse ME microtome was used to section unstained femur sections that were 4 µm thick before they were examined under fluorescence microscopy prior to calcein double labeling analysis. The average width along with the labeling period (days between injections of 10 μg/g calcein intraperitoneally at 7 days and 2 days before euthanasia) was then used to calculate the mineralizing surface (MS/BS, %), bone formation rate (BFR, μm^3^μm^-2^ per day) and mineral apposition rate (MAR, μm per day).

L3-L5 vertebrae were fixed in 4% paraformaldehyde for 48 h and subjected to 48 hours of exposure to 70% ethanol before being decalcified in 10% EDTA for 14-21 days. The processed samples were finally paraffin-embedded, sectioned at a thickness of 4 µm, and stained by hematoxylin-eosin staining (H&E) and tartaric acid-resistant acid phosphatase staining (TRAP). The bone histomorphometry parameters, including osteoblast surfaces (Ob.S/BS, %), number of osteoclasts (N.Oc/B.Pm, /mm) and osteoclast surfaces (Oc.S/BS, %), were analyzed by an investigator blinded to sample collection and group assignment using an OsteoMeasure Image Analysis System (Osteometrics, Decatur, GA).

### Enzyme-linked immunosorbent assay (ELISA)

TRACP-5b and osteocalcin serum concentrations were quantified using R&D Systems IDS (Fountain Hills, AZ, USA) ELISA kits. Cheek pouch punctures were done on mice fasted for 4 hours for blood samples. The samples were then assessed through 450 nm absorbance.

### Statistical analysis

SPSS statistics version 19.0 (IBM, Chicago, IL, USA) was used to analyze the data. All data were normally distributed, with similar variances between the experimental groups. Two sample groups were assessed using the two-tailed Student's *t* test, while groups with more than two samples were assessed using one-way ANOVA and two-way ANOVA tests if there was one or more than two conditions, respectively. The *post hoc* Bonferroni's correction test was conducted following each ANOVA analysis for multiple comparisons. Statistical significance was derived when the *P* value was less than 0.05. ^*^P value < 0.05 and ^**^P value < 0.01. Data were depicted in terms of mean ± SD or ± SEM as indicated in the figure legends.

## Results

### High miR-128 expression is correlated with increased bone resorption

**Figure 1:**
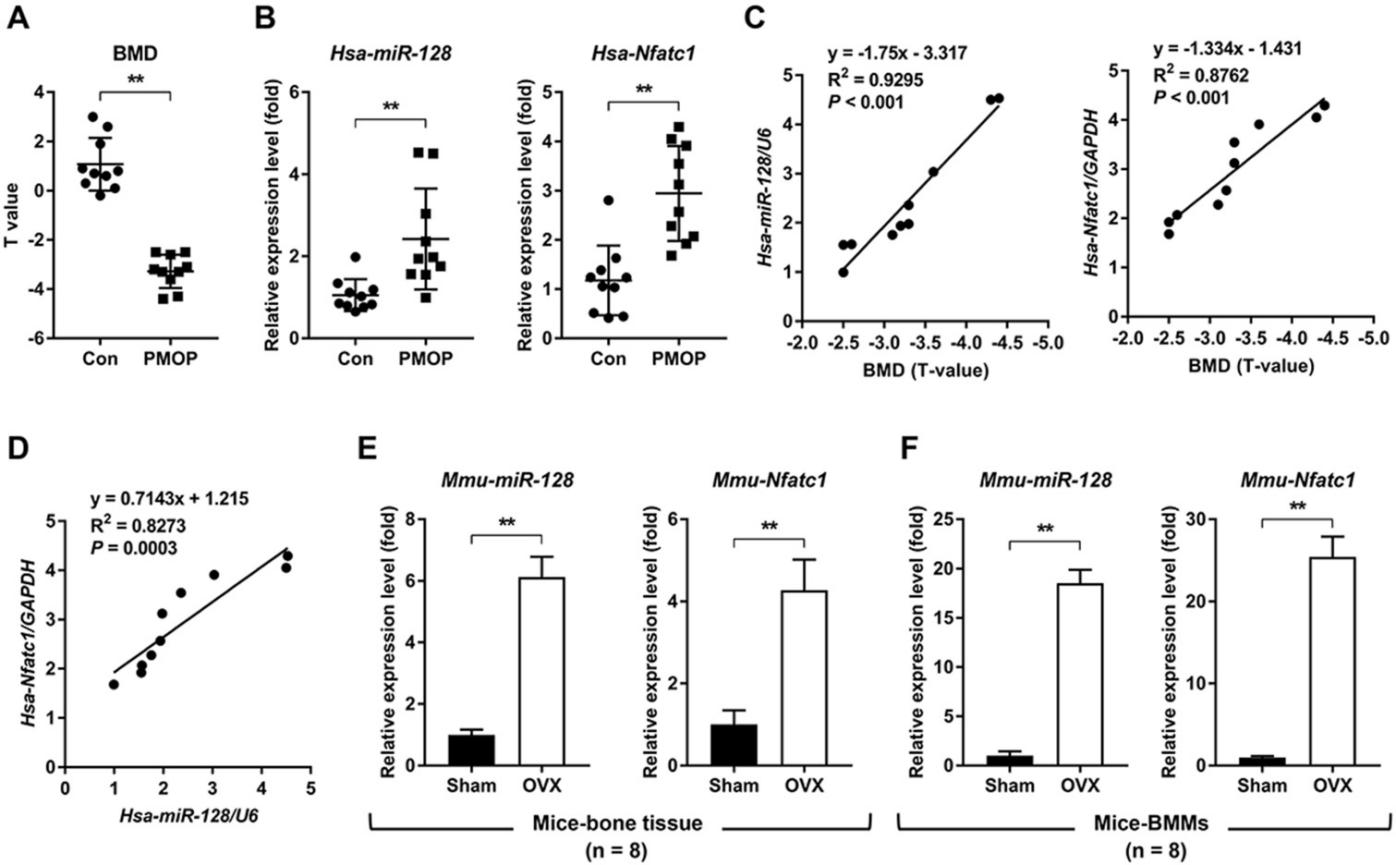
** miR-128 levels positively correlate with increased bone resorption in mouse/human bone specimens and mouse primary BMMs. A** BMD of the control group and the PMOP group. Mean ± SD, n = 10 biologically independent samples, ^**^P < 0.01 by Student's *t*-test. **B** qRT-PCR analysis showed that *miR-128* and *Nfatc1* mRNA expression levels significantly increased in PMOP patients-derived bone tissues. Mean ± SD, n = 10 biologically independent samples, ^**^P < 0.01 by Student's *t*-test. **C** Correlation analysis demonstrated that the expression levels of *miR-128* and* Nfatc1 mRNA* increased along with the decrease in BMD levels in PMOP patients. n = 10 biologically independent samples. **D** Correlation analysis demonstrated that *miR-128* expression levels are positively correlated with increased *Nfatc1* mRNA levels in bone tissues from PMOP patients. n = 10 biologically independent samples. **E, F** qRT-PCR analysis showed that *miR-128* and *Nfatc1* mRNA expression levels significantly increased in OVX mice-derived bone tissues and BMMs. Mean ± SD, n = 8 biologically independent samples, ^**^P < 0.01 by Student's *t*-test.

To investigate the relationship between miR-128 level and human PMOP, bone tissues from patients with or without PMOP (Figure 1A) were subjected to qRT-PCR. Compared with the control group, the PMOP group showed markedly increased expression levels of *miR-128* and the master osteoclastic transcription factor *Nfatc1* (Figure 1B). Addtionally, the expression levels of *miR-128* and *Nfatc1* increased along with the decreasing BMD levels in PMOP patients (Figure 1C). And *miR-128* expression levels were positively correlated with *Nfatc1* expression levels in bone tissues of PMOP patients (Figure 1D). Furthermore, we examined the expression levels of *miR-128* in the vertebrae and BMMs derived from the sham and OVX mice. Compared with the sham group, the OVX group exhibited significantly enhanced in *miR-128* levels in vertebrae and BMMs (Figure 1E-F).

**Figure 2:**
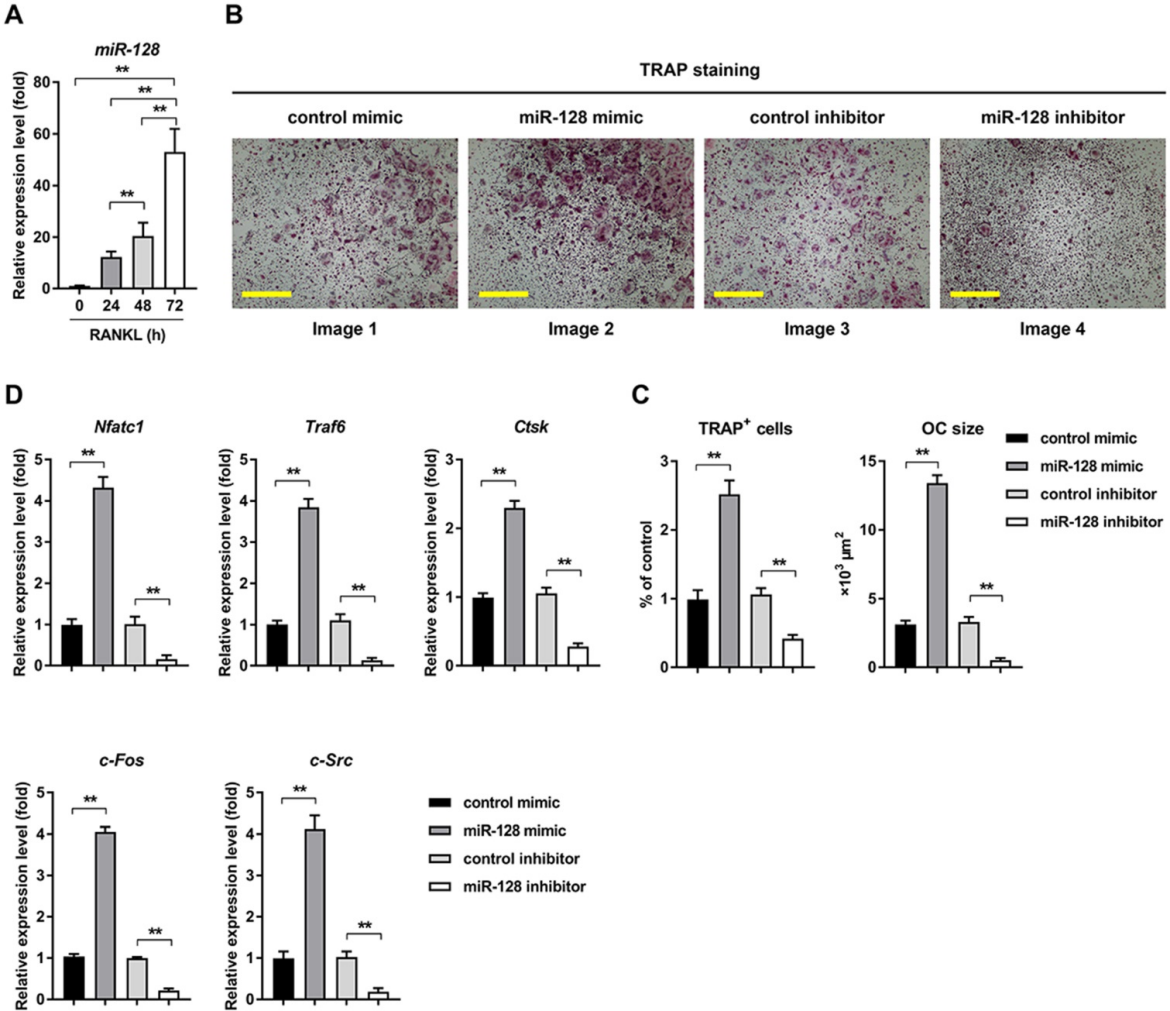
** miR-128 is an important regulator of osteoclastogenesis in BMMs. A** Mature mmu-miR-128 expression upon RANKL-induced osteoclastogenesis. Data is depicted in terms of mean ± SD. ^**^P < 0.01 by one-way ANOVA with Tukey' s *post hoc* test. **B** RANKL-induced osteoclast differentiation for three days in transfected BMMs containing either miR-128 mimic/inhibitor or control mimic/inhibitor. TRAP staining was performed. Scale bars: 200 μm. **C** Osteoclast size and number. TRAP-positive cells with at least three nuclei were designated to be osteoclasts. Data is depicted in terms of mean ± SD. ^**^P < 0.01 by one-way ANOVA with Tukey' s *post hoc* test. **D** qRT-PCR of mRNA expression of *Nfatc1*, *Traf6*, *Ctsk*, *c-Fos*, and *c-Src*. Data is depicted in terms of mean ± SD. ^**^P < 0.01 by one-way ANOVA with Tukey' s *post hoc* test.

In response to RANKL treatment, miR-128 expression level increased with time course (0h, 24h, 48h, and 72h) during osteoclast differentiation (Figure 2A). The *in vitro* relationship between miR-128 and osteoclast differentiation was also explored. Overexpression of miR-128 caused by a miRNA mimic (the efficiency of miR-128 overexpression was shown in Supplementary Figure 1A) in the BMMs resulted in significantly increased osteoclastogenesis, as evidenced by TRAP staining (Figure 2B, images 1 vs. 2). Quantitative analyses confirmed increases in number and size in the TRAP positive cells (Figure 2C, columns 1 vs. 2). Moreover, qRT-PCR analysis uncovered augmented mRNA levels of *Nfatc1*, as well as *Traf6*, *Ctsk*, *c-Fos*, and *c-Src* in miR-128 mimic treated BMMs when contrasted to the controls (Figure 2D, columns 1 vs. 2). We also used a specific miR-128 inhibitor (the efficiency of miR-128 knockdown was shown in Supplementary Figure 1B) to assess BMMs exposed to RANKL. Conversely, downregulation of miR-128 in BMMs contributed to significantly decreased osteoclast differentiation (Figure 2B, images 3 vs. 4; Figure 2C, columns 3 vs. 4) and the osteoclastic gene expressions of *Nfatc1*, *Traf6*, *Ctsk*, *c-Fos*, and *c-Src* (Figure 2D, columns 3 vs. 4). There was no significant differences in proliferation or apoptosis of BMMs found between the miR-128 inhibitor/mimic and control group (Supplementary Figure 2A). These results indicated that miR-128 may be a positive player in osteoclast differentiation.

### miR-128 controls bone homeostasis by regulating osteoclastogenesis

**Figure 3:**
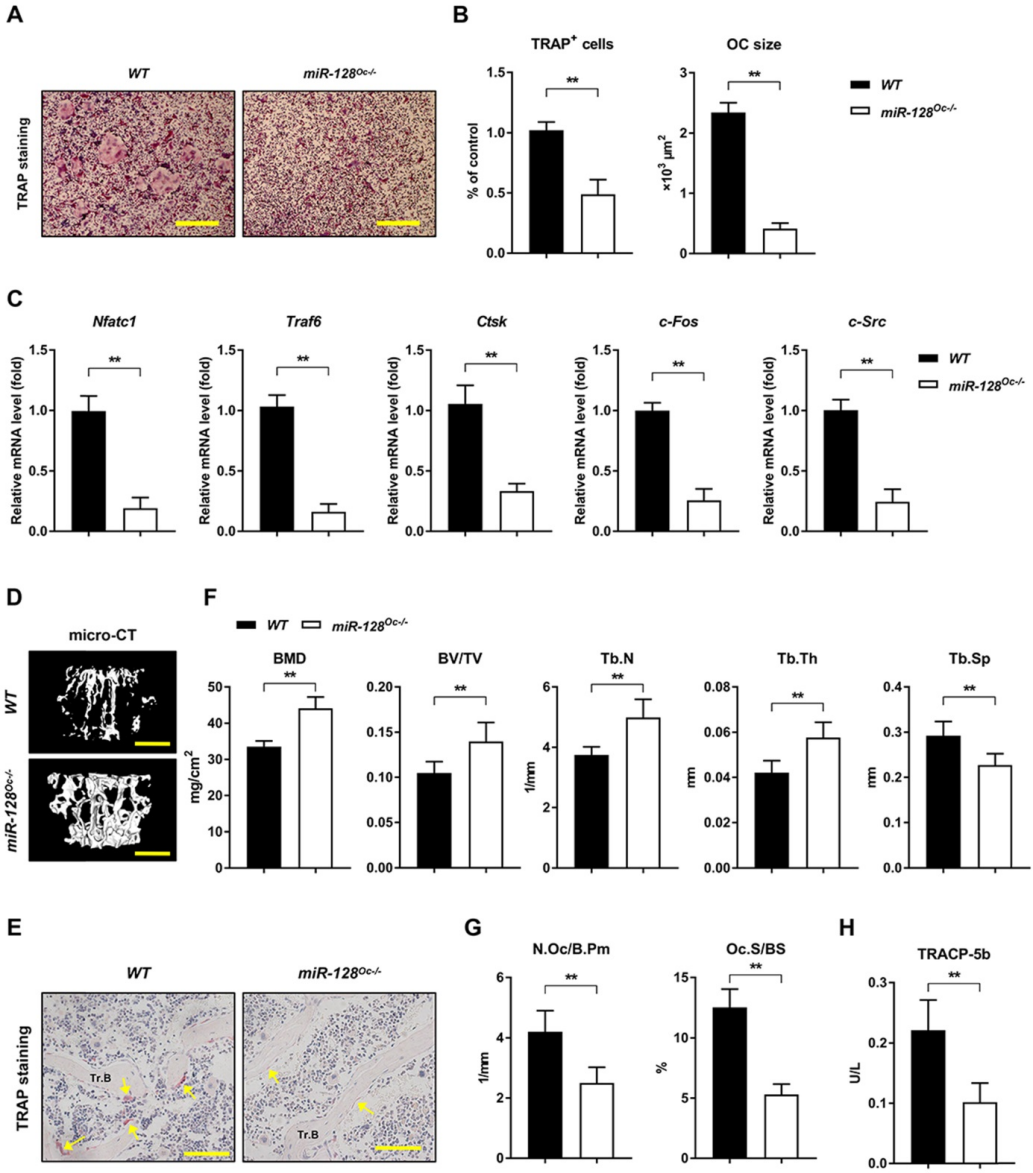
** miR-128 controls bone homeostasisis via the regulation of osteoclastogenesis. A** Osteoclast differentiation of BMM harvested from *WT* and *miR-128^Oc-/-^* mice. TRAP staining was carried out. Scale bars: 200 μm. **B** Osteoclast number and size. TRAP-positive cells with a minimum of three nuclei were designated to be osteoclasts. Data are mean ± SD. ^**^P < 0.01 by Student's *t* test. **C** Quantitative real-time PCR analysis of mRNA expression of *Nfatc1*, *Traf6*, *Ctsk*, *c-Fos*, and *c-Src*. Data are mean ± SD. ^**^P < 0.01 by Student's *t* test. **D** Representative images of micro-CT reconstruction (Scale bars: 500 μm) and **E** representative TRAP-stained sections (yellow arrows: osteoclasts) of trabecular bone (Tr.B) of the L1 vertebrae isolated from 10-week-old littermate male *WT* and *miR-128^Oc-/-^* mice (n = 8/group) (Scale bars: 100 μm). **F, G** Histomorphometry analysis of the metaphysis region of the L1 vertebrae isolated from 10-week-old littermate male *WT* and *miR-128^Oc-/-^* mice. Data are mean ± SEM. ^**^P < 0.01 by Student's *t* test. **H** Serum TRACP-5b values tested by ELISA from *WT* and *miR-128^Oc-/-^* mice. Data are presented as mean ± SEM. ^**^P < 0.01 by Student's *t* test.

The miR-128 was specifically deleted in BMMs by crossing *miR-128^flox/flox^* mice with *LysM-Cre* mice to produce *miR-128^Oc-/-^* mice, which were first established. The knockout efficiency of *miR-128^Oc-/-^* mice and *WT* mice are shown in Supplementary Figure 1C. The *miR-128^Oc-/-^* mice were analogous from the *WT* mice in appearance at birth and later in life (Supplementary Figure 1D)*.* In contrast to *WT* cell cultures, the deficiency in miR-128 in the *miR-128^Oc-/-^* BMMs cultures dramatically decreased osteoclastogenesis, as evidenced by TRAP staining (Figure 3A, B). In parallel with the suppressed osteoclast differentiation, the expression of crucial osteoclastogenic molecules *Nfatc1*, *Traf6*, *Ctsk*, *c-Fos*, and *c-Src* was markedly decreased in *miR-128^Oc-/-^* mice-derived BMMs in relation to the *WT* control cells (Figure 3C). This suggests that osteoclastogenesis is strongly affected by miR-128 levels. The bony phenotype of the osteoclastic miR-128 knockout mice was then assessed via micro-CT analyses. The findings indicate a strong bone protection phenotype in these *miR-128^Oc-/-^* mice, as evidenced by decreased Tb.Sp and increased BMD, Tb.Th, Tb.N, BV/TV and trabecular bone mass, in contrast to mice in the control cohort (Figure 3D, F). Furthermore, miR-128 appears to have an *in vivo* effect on the construction of osteoclasts, as shown by suppressed levels of osteoclasts in the *miR-128^Oc-/-^* mice in comparison with *WT* controls (Figure 3E, G). Serum markers of bone turnover was then assessed with ELISA assays. Significantly lower levels of TRACP-5b, an indicator of bone resorption, was found in the *miR-128^Oc-/-^* mice (Figure 3H). There were no significant differences in proliferation or apoptosis of BMMs between the *miR-128^Oc-/-^* and *WT* group (Supplementary Figure 2B). Additionally, osteoclastic deficiency in miR-128 did not impact N.Ob/B.Pm, BFR, MAR, MS/BS and osteocalcin, serum levels of the bone formation marker. Furthermore, no significant differences were observed in ALP staining, ARS staining, ALP activity, and calcium mineralization, as well as the expression levels of osteogenic specific genes (*Runx2*,* Sp7*,* Alp*, and* Ocn*) in *miR-128^Oc-/-^* mice-derived osteoblastic stromal cells compared with the *WT* control cells under osteogenic induction (Supplementary Figure 3D-G). Overall, our data highlighted miR-128 as a critical modulator of bone homeostasis via the regulation of osteoclastogenesis.

### miR-128 regulates osteoclastogenesis by targeting SIRT1 and NF-κB signaling

**Figure 4:**
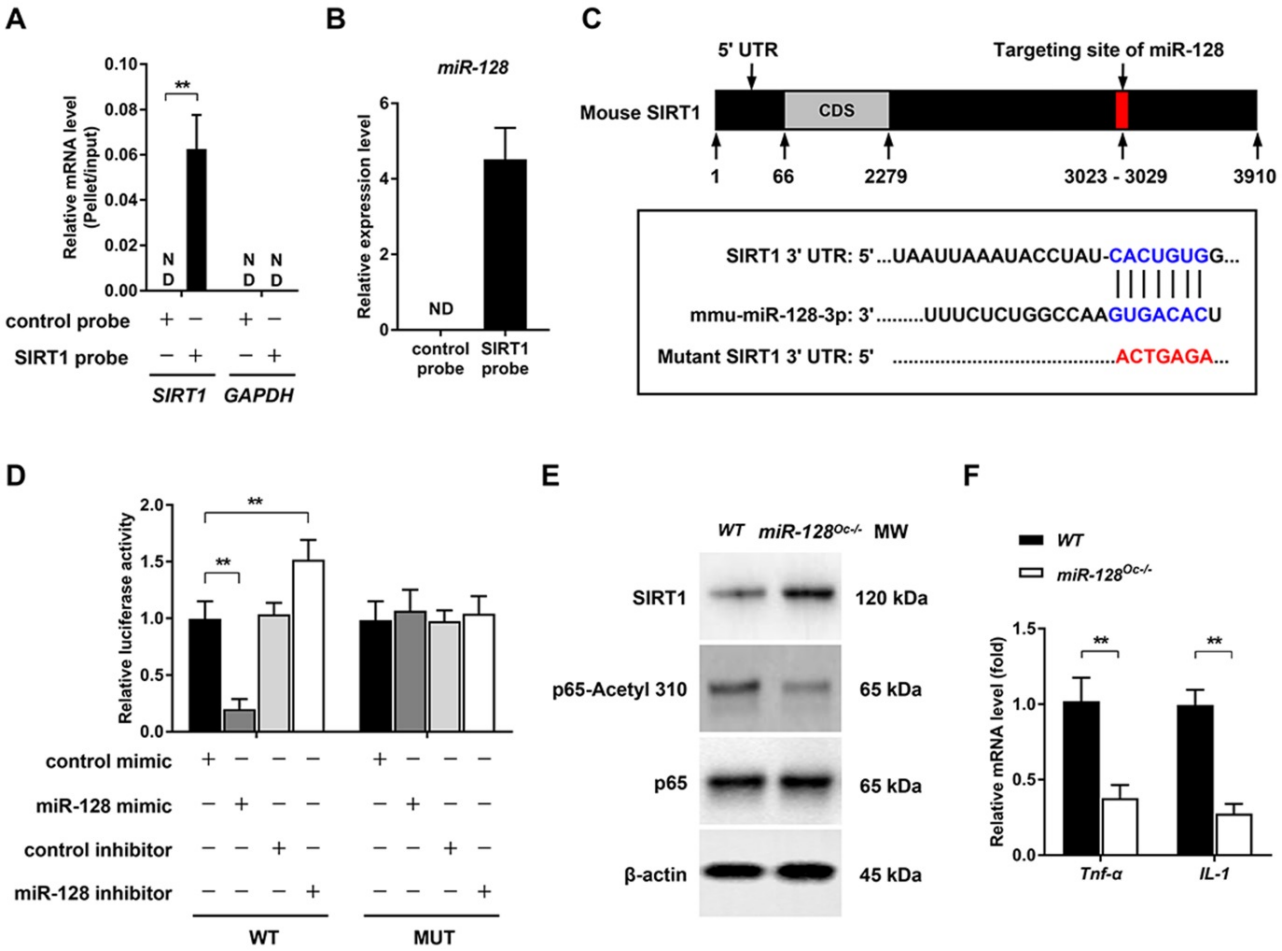
** miR-128 regulates osteoclastogenesis by targeting SIRT1 and NF-κB signaling. A** Efficiency and specificity of the SIRT1 probe by qRT-PCR. Data are presented as mean ± SD. ^**^P < 0.01 by two-way ANOVA. **B** miR-128 could bind to SIRT1 mRNA, as shown by pull down assays. The expression of miR-128 was detected by qRT-PCR. Data are presented as mean ± SD. ^**^P < 0.01 by Student's *t* test. **C** Schematic illustrations of the hypothetical duplexes formed by miR-128 with the 3' UTR of SIRT1. **D** Luciferase activities as quantified from the BMMs co-transfected with the *WT* or mutant 3' UTR of SIRT1 luciferase reporter plasmids together with miR-128 inhibitor or mimic and or the corresponding control. Data are presented as mean ± SD. ^**^P < 0.01 by two-way ANOVA. **E** Western blot analysis of SIRT1, p65-Acetyl 310, and p65 protein levels in BMMs derived from *WT* and *miR-128^Oc-/-^* mice. **F** mRNA expressions of *Tnf-α* and *IL-1* in BMMs derived from *WT* and *miR-128^Oc-/-^* mice were analysed via qRT-PCR analysis. Data are presented as mean ± SD. ^**^P < 0.01 by Student's *t* test.

Several biological processes are subjected to miRNA regulation, which works by altering the expressions of target genes. Given that we have shown the essential role of miR-128 in osteoclastogenesis, we aimed to further clarify the target genes of this miRNA. By the bioinformatics analysis, SIRT1 was found to be a candidate of great interest. Previous studies have demonstrated that miR-128 is able to aggravate Tnf-α-induced inflammatory response, a physiopathological process that is associated with osteoclastogenesis and osteoporosis 27, 28, via the regulation of SIRT1 in BMSCs 16. Moreover, SIRT1 is known as a key negative player in osteoclastogenesis and osteoporosis 29. For instance, specific SIRT1 deficiency in murine osteoclast (*SIRT1^flox/flox^*_*LysM-Cre*, the genetic approach used is consistent with our study) was found to activate osteoclastogenesis by promoting NF-κB signaling *in vitro* and results in osteoporosis in mice 30.The relationship between SIRT1 and miR-128 was tested using a pull-down assay, where a biotinylated SIRT1 probe was used to pull down SIRT1 mRNA. miR-128 expression levels were then quantified with qRT-PCR in pellets containing SIRT1 mRNA (Figure 4A). Figure 4B showed that miR-128 bound directly to SIRT1 mRNA, as evidenced by detection of miR-128 in the pellet. As predicted by Targetscan, miR-128 have a conserved binding site in the 3' UTR of SIRT1 (Figure 4C). To determine whether miR-128 could inhibit SIRT1 expression, BMMs derived from *miR-128^Oc-/-^* and *WT* mice were used to test the SIRT1 protein levels. As anticipated, SIRT1 levels were markedly enhanced in miR-128 deficient-BMMs in contrast to cells derived from *WT* mice (Figure 4E and Supplementary Figure 4A). Luciferase assays were carried out to predict the seed sequence binding sites responsible for the miRNA-mRNA interaction. DNA fragments that containing the miR-128 binding sites of SIRT1 3' UTR were inserted into the pMIR-Report Luciferase vector. BMMs were then co-transfected with the aforementioned vector, miR-128 mimic/inhibitor. Figure 4D then demonstrated that the ectopic miR-128 expression markedly suppressed the activity of luciferase, while suppression of miR-128 promoted fluorescence intensity. A mutant luciferase vector that contained a miR-128 binding site in the SIRT1 3' UTR, which was able to inhibit the interaction of miR-128 and SIRT1 mRNA, was constructed. The mutant plasmid was used to repeat the luciferase experiments, which resulted in the miR-128 mimic or inhibitor no longer being able to influence luciferase activity. These results suggested that the inhibition of SIRT1 occurs as a result of miR-128 binding to its 3' UTR.

Previously, the deletion of SIRT1 in osteoclasts (*SIRT1^fl/fl^_LysM-Cre*) has been reported to promote osteoclastogenesis and activate NF-κB by augmenting Lysine 310 acetylation, a significant regulator of bone resorption and the formation of osteoclasts 30. In this context, we examined whether NF-κB signaling was regulated by miR-128. We found that the acetylation levels of lysine 310 of the p65 subunit of NF-κB had significantly decreased in *miR-128^Oc-/-^* BMMs versus *WT* controls (Figure 4E and Supplementary Figure 4A). Also, miR-128 deficiency in osteoclast remarkedly inhibited the mRNA expression of *Tnf-α* and *IL-1* (Figure 4F). Next, we focused on miR-128 to explore the consequences of miR-128-driven SIRT1 suppression and NF-κB activation in osteoclastogenesis.

**Figure 5:**
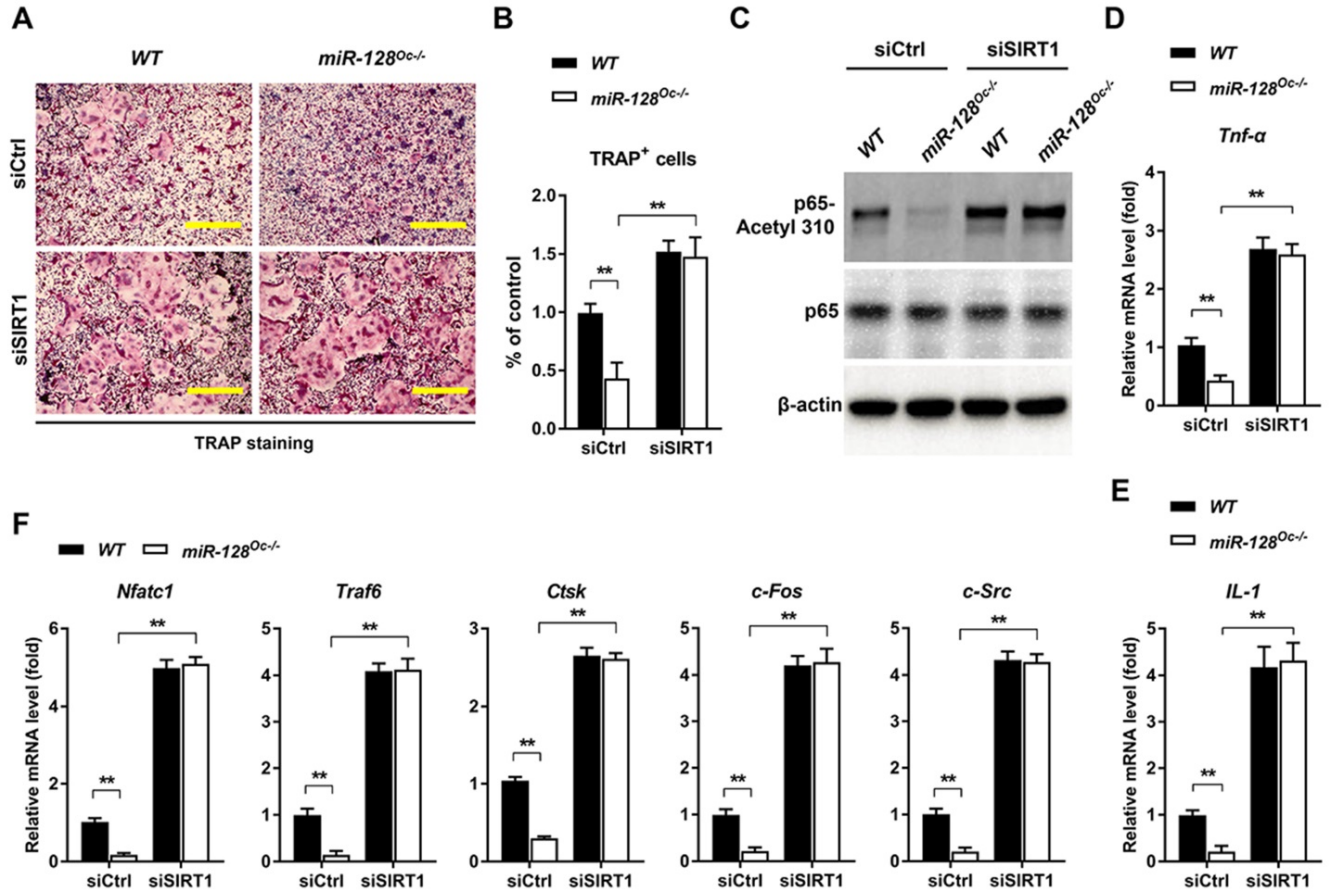
** The decrease of osteoclastogenesis by osteoclastic miR-128 deficiency was due to its inhibitory effect on SIRT1. A** Osteoclast differentiation as observed in BMMs derived from *WT* and *miR-128^Oc-/-^* mice that were transfected with the control siRNAs (siCtrl) or siRNA-targeted SIRT1 (siSIRT1) and stimulated with RANKL for three days. TRAP staining was performed. Scale bars: 200 μm. **B** Osteoclast number and size. TRAP-positive cells with at least three nuclei were counted as osteoclasts. Data are presented as mean ± SD. ^**^P < 0.01 by two-way ANOVA. **C** Western blot analysis of p65-Acetyl 310 and p65 protein levels in BMMs derived from *WT* and *miR-128^Oc-/-^* mice that were transfected with the siCtrl or siSIRT1. **D, E** qRT-PCR analysis of mRNA expression of *Tnf-α* and *IL-1* in BMMs derived from *WT* and *miR-128^Oc-/-^* mice that were transfected with the siCtrl or siSIRT1. Data are presented as mean ± SD. ^**^P < 0.01 by two-way ANOVA. **F** qRT-PCR analysis of mRNA expression of *Nfatc1*, *Traf6*, *Ctsk*, *c-Fos*, and *c-Src* in BMMs derived from *WT* and *miR-128^Oc-/-^* mice that were transfected with the siCtrl or siSIRT1. Data are presented as mean ± SD. ^**^P < 0.01 by two-way ANOVA.

To examine whether the suppression of osteoclastogenesis by miR-128 deficiency was due to the targeting of SIRT1 and NF-κB signaling by miR-128, we performed recovery experiments in which a SIRT1-targeted siRNA (siSIRT1) was used to specifically reverse osteoclastic miR-128 knockout- mediated increase of SIRT1 expression (the efficiency of the siRNA SIRT1 is shown in Supplementary Figure 1E). As shown in Figure 5A and B, knockdown of SIRT1 in BMMs markedly abolished the osteoclastic inhibition effect of miR-128 deficiency, as evidenced by TRAP staining. There were no significant differences in proliferation or apoptosis of the BMMs observed between the *miR-128^Oc-/-^* and *WT* groups after siSIRT1 treatment (Supplementary Figure 2C). Furthermore, siSIRT1 treatment evidently enhanced the lysine 310 acetylation levels of the p65 subunit of NF-κB (Figure 5C and Supplementary Figure 4B), *Tnf-α* and *IL-1* levels (Figure 5D, E), as well as osteoclastic gene expressions (*Nfatc1*, *Traf6*, *Ctsk*, *c-Fos*, and *c-Src*) that had been inhibited by miR-128 deficiency in the BMMs (Figure 5F). As a whole, these results highlight that miR-128 acted as a pro-osteoclastogenesis miRNA to activate osteoclast differentiation by targeting SIRT1 and subsequently activating NF-κB by increasing acetylation of Lysine 310.

### Deletion of miR-128 in osteoclasts prevents OVX-induced bone loss

**Figure 6:**
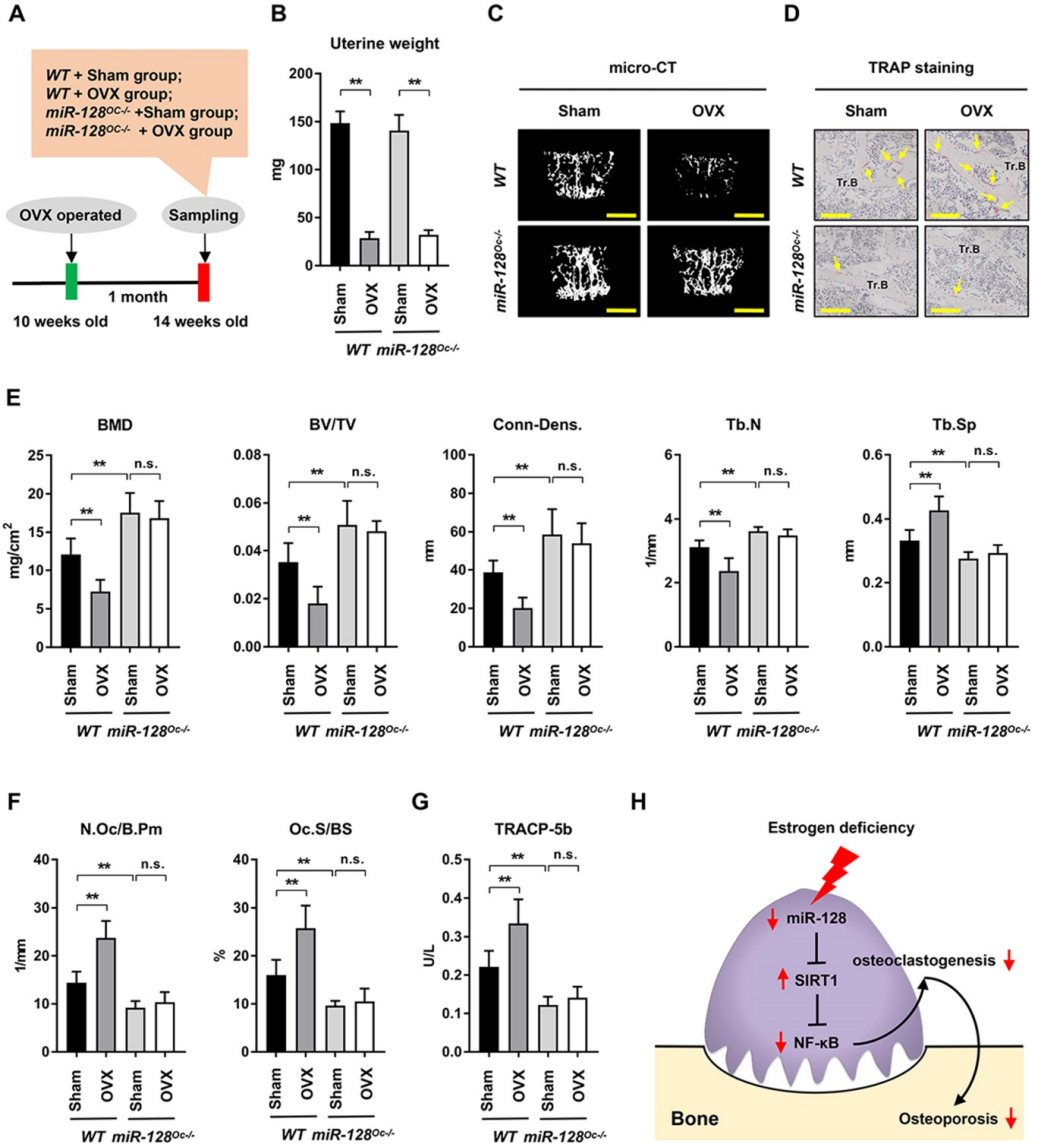
** Osteoclastic miR-128 deficiency prevents mice from OVX-induced bone loss. A** A schematic diagram depicting the procedural steps. **B-G** OVX or sham surgery was done on 10-week-old female *WT* and *miR-128 miR-128^Oc-/-^* mice. Mice were then analysed at 5 weeks post-procedure. **B** Uterine weights of *WT* and *miR-128^Oc-/^* mice with sham or OVX surgery. Data is depicted in terms of mean ± SEM. ^**^P < 0.01 by two-way ANOVA. **C** Representative images of micro-CT reconstruction (Scale bars: 500 μm) and **D** representative TRAP-stained sections (yellow arrows: osteoclasts) of Tr.B of the L1 vertebrae harvested from the *WT* and *miR-128^Oc-/-^* mice with sham or OVX surgery (n = 8/group) (Scale bars: 200 μm). **E, F** Histomorphometry analysis of the L1 vertebrae metaphyseal region dissected from 10-week-old littermate male *WT* and *miR-128^Oc-/-^* mice with sham or OVX surgery. Data is depicted in terms of mean ± SEM. ^**^P < 0.01 by two-way ANOVA. **G** Serum TRACP-5b values tested by ELISA from 10-week-old littermate male *WT* and *miR-128^Oc-/-^* mice with sham or OVX surgery. Data is depicted in terms of mean ± SEM. ^**^P < 0.01 by two-way ANOVA. **H** Model of the novel identified miR-128/SIRT1/NF-κB axis in osteoclastogenesis and osteoporosis.

The impact of miR-128 in OVX-induced bone loss was also studied. With the premise that estrogen deficiency is the most significant contributor towards bone loss 3, OVX models were established in *miR-128^Oc-/-^* mice to determine the role of osteoclastic miR-128 in estrogen deficiency induced osteoporosis, a condition that is highly similar to postmenopausal bone loss (Figure 6A). The uterine weight of OVX mice were assessed 5 weeks after the procedure to assess its efficiency. OVX mice contained uteruses that weighted 75% less than mice with intact ovaries (Figure 6B), indicating effective estrogen depletion. OVX markedly depleted bone mass as evidenced by a 39.95% decrease in BMD, 48.83% decrease in BV/TV, 47.99% decrease in Conn-Dens., 23.89% decrease in trabecular number, and 28.59% increase in trabecular spacing in contrast to the control groups (Figure 6C and E, columns 1 vs. 2), as demonstrated by the micro-CT analysis. Interestingly, although *miR-128^Oc-/-^* mice had markedly higher bone mass at the unaltered state (Figure 6C and E, columns 1 vs. 3), we observed a less obvious decline in BMD, BV/TV, Conn-Dens., Tb.N, and Tb.Th, as well as a less obvious increase in Tb.Sp in *miR-128^Oc-/-^* mice after OVX, compared with the controls (Figure 6C and E, columns 3 vs. 4). TRAP staining, bone histomorphometry analysis and ELISA assay showed that there was a marked increase in the numbers and sizes of osteoclasts and serum levels of TRACP-5b in the *WT* OVX group, compared to the *WT* sham group (Figure 6D and F, G, columns 1 vs. 2). Conversely, in the sham group, miR-128 deficiency in osteoclasts reduced levels of bone resorption parameters (Figure 6F, G, columns 1 vs. 3), while no significant difference was observed in bone resorption parameters between the *miR-128^Oc-/-^* sham group and *miR-128^Oc-/-^* OVX group (Figure 6F, G, columns 3 vs. 4). However, osteoclastic miR-128 deficiency did not affect osteoblastic parameters, including osteoblast surfaces, MS/BS, MAR, and BFR, in addition to serum levels of osteocalcin in the sham and OVX mice (Supplementary Figure 5). Our findings put forth that osteoclastic deletion of miR-128 appears to prevent OVX-induced pathological bone loss.

## Discussion

PMOP mainly occurs in postmenopausal women and is a common disease associated with aging 2, 31. It has recently been reported that regulation of miR-128 expression could be used to modulate cell senescence and age-related pathological conditions such as Alzheimer's disease (AD), cardiovascular diseases, cancer, and the aging process. Lan's research group suggested that the upregulation of miR-128 was found in the blood vessels of old individuals and old mice, as well as in senescent human umbilical vein endothelial cells (HUVECs) 32. A clinical study also suggested that augmentation of miR-128 levels corresponded with the aberrant degeneration of monocytic amyloid β (1-42) in patients with sporadic AD 33. Other related studies also reported of the high expression of miR-128 in aged hippocampus compared to that at the fetal and adult stages 34. An integromics network meta-analysis suggested that miR-128/-27b demonstrated significant synergy and their association to cardiovascular and age-related condition is echoed in several studies on population- based disease databases 35. Noren et al. identified miR-128 to be involved in the progression of cancer, as suppressed levels were found in elderly patients. In addition, its predicted targets such as PI3 kinase (PI3K), c-Kit and H2AX, were also discovered to have increased in aging individual, highlighting its significance in the process of aging 36. miR-128 expression was also found to be suppressed in the kidneys of old *WT* and Ercc1^-/Δ^ mice in comparison with young *WT* mice as well as in liver samples of old *WT* and progeroid Ercc1^-/Δ^ mice 37. These studies highlight the important role of miR-128 in age-related diseases, such as PMOP 38, 39. Further, Zhao et al. reported that the mRNA expression of miR-128 in the PMOP group was remarkably higher than that in the non-PMOP group 19. In line with these findings, our experiments revealed that postmenopausal women possessed higher levels of both the osteoclastic marker gene *Nfatc1* and *miR-128*. Moreover, a correlation analysis demonstrated that miR-128 expression levels are positively correlated with increased *Nfatc1* mRNA levels in bone tissues from PMOP patients. miR-128 may be mechanistically involved in the biological process of bone resorption, propelling the progression from osteopenia to osteoporosis. This underscores its potential as a marker of osteoclastogenesis. These findings warrant validation using studies with bigger cohort sizes.

Chronic inflammation is a prominent feature in PMOP 10, 40, 41 and is marked by the increased production of pro-inflammatory cytokines which comprise of tumor necrosis factor (TNF), interleukin 1 (IL-1), and IL-6, all of which augment RANKL expression 38, 42-44. Previously, miR-128 was reported to participate in the regulation of inflammatory related diseases, such as RA 15 and OA 18, inflammatory signaling, like NF-κB 15 and TNF-α 16, and ox-LDL-incubated RAW264.7 17, which raises the possibility that miR-128 participates in the regulation of osteoclastogenesis and osteoporosis. Here, using miR-128 overexpression and knockdown approaches, we first reported that miR-128 enhances *in vitro* differentiation of osteoclasts. More importantly, we established that miR-128 deficiency in BMMs increases bone mass results from the inhibition of osteoclastogenesis. In addition, numerous researchers studies have revealed that miR-128 is an important osteogenesis-suppressive miRNA in different cell types, though the potential mechanism of miR-128 in regulating osteoblastogenesis is still unclear. In C2C12 cells, miR-128 suppresses osteogenic differentiation via targeting SIRT6 19. For human MSCs, miR-128 expression was found to increase upon adipogenic treatment and decease upon osteogenic treatment 45. Overexpression of miR-128 inhibits vascular endothelial growth factor (VEGF), thus suppressing osteogenic differentiation and promoting adipogenic differentiation 46. Nevertheless, in our study, compared to the strong osteoclastic impact, inhibition of miR-128 in BMMs failed to affect osteoblast activity* in vitro*. We conclude that this may be due to specific ablation of miR-128 in BMMs. To our knowledge, PMOP is mainly a result of increase in bone resorption, while age-related osteoporosis is mainly caused by the decline of bone formation 47, 48. Therefore, we assume that investigation of the role of miR-128 in osteoblastogenesis and age-related osteoporosis by generating osteoblastic miR-128 knockout or transgenic mice (e.g. created by breeding *miR-128^flox/flox^* mice with a *Prx1-Cre* mice) will require further studies.

miRNAs are key modulators of a plethora of biological processes given their ability to modulate several different gene targets. Exploration of their different functions are crucial in gaining deeper insights into the pathophysiology of several diseases. miRNAs target different genes through complementary seed region binding. miRNAs may regulate the same target gene differently based on the cellular microenvironment due to its diversity of genetic expression and subsequent molecular profile of each cell under different stressors and conditions 49-53. These experiments identified SIRT1 as a new target for miR-128 during osteoclast differentiation. SIRT1 appears to be the key target of miR-128 upon differentiation of osteoclasts as proven by experiments demonstrating the suppression of miR-128 function upon silencing of SIRT1. SIRT1 is a deacetylase dependent on nicotinamide adenine dinucleotide (NAD) that works to slow the aging process in less developed organisms and countereffects the progression of age-related diseases in mammals 54-56. SIRT1 modulates processes such as mitochondrial homeostasis, tumor suppression, energy metabolism and repair of DNA 57, 58. The role of SIRT1 in modulating osteoclasts-dependent bone resorption and osteoporosis has been recognized and was recently reviewed by Zainabadi 29. These previous studies, including monogenic bone disorders and knockout mice, confirmed that SIRT1 is a key negative player in osteoclastogenesis and osteoporosis. Similar to our genetic approach that used LysM-Cre to induce recombination in monocytes, Florent's group 27 demonstrated that osteoclast specific SIRT1 deletion by crossing the *SIRT1^flox/flox^* mice to *LysM-Cre* transgenic mice leads to lower bone mass as a result of enhanced bone resorption. Mechanistically, deletion of SIRT1 in osteoclasts enhances osteoclastogenesis *in vitro* and causes NF-κB activation through enhanced acetylation of Lysine 310. This augmentation in osteoclast differentiation is abolished by the pharmacological inhibition of NF-κB signaling. In this study, our data suggested that miR-128 directly targets SIRT1 at the post-transcriptional level during osteoclast differentiation. The increased SIRT1 reduced NF-κB activity by decreasing acetylation of Lysine 310 in *miR-128^Oc-/-^* BMMs, as well as suppressed *Tnf-α* and *IL-1* expressions. Recovery experiments also mirrored these results. Our findings uncovered a new driver of osteoclastogenesis, the miR-128/SIRT1/NF-κB signaling axis and identified miR-128 as a novel therapeutic target for osteoporosis.

In this study, our data identified miR-128 as a new osteoclastogenesis and bone remodeling modulator. Estrogen deficiency is the main driver of PMOP, a condition resulting in several fractures in elderly women. We were intrigued to find that osteoclastic miR-128 deficiency also ameliorated OVX-induced bone loss. Our findings showed that miR-128 loss-of-function in osteoclasts markedly ameliorated trabecular bone loss in OVX mice, indicating that miR-128 has a physiological effect on bone metabolism. Alterations in the number and size of osteoclasts and TRACP-5b serum levels indicate that the therapeutic effect of *miR-128^Oc-/-^* may be due to the inhibition of bone resorption. Recently, some new molecules has been utilized in the development of the treatment strategies for osteoporosis 59. Denosumab, a RANKL monoclonal antibody that suppresses excessive bone resorption is a strong example 60. miR-128 appears to be a powerful stimulator of RANKL-induced osteoclast generation, highlighting its role in the development of novel PMOP treatments.

## Conclusion

In summary, we show that miR-128 levels positively correlate with increased bone resorption in mouse/human bone specimens and mouse primary BMMs. *In vitro*, miR-128 knockdown in BMMs inhibits osteoclast differentiation, while its overexpression shows negative results. *In vivo*, osteoclastic miR-128 knockout shows a strong protection in mice and suppresses osteoclastogenesis. This effect is mainly through the increased in the expression of SIRT1 at the post-transcriptional level, which subsequently inactivates NF-κB signaling through reducing the acetylation of Lysine 310, suggesting that the regulatory pattern of the miR-128/SIRT1/NF-κB signaling axis might be a critical mechanism for osteoclast differentiation. Further, osteoclastic miR- 128 deletion prevents estrogen deficiency- induced bone loss in mice (Figure 6H). Our data demonstrate for the first time that miR-128 is a crucial regulator of murine osteoclastogenesis, and indicates its utility as a diagnostic and therapeutic target for PMOP.

## Supplementary Material

Supplementary figures and table.

## Abbreviations

miR-128: microRNA-128
Nfatc1: nuclear factor of activated T cells 1
BMMs: bone marrow-derived macrophages
OVX: ovariectomy
SIRT1: sirtuin 1
NF-κB: nuclear factor κB
Tnf-α: tumor necrosis factor-α
IL-1: interleukin 1
M-CSF: macrophage colony-stimulating factor
RANKL: receptor activator of NF-κB ligand
TRAF6: TNF receptor-associated factor 6
RA: rheumatoid arthritis
TNFAIP3: tumor necrosis factor-α-induced protein 3
BMSCs: bone mesenchymal stem cells
ox-LDL: oxidized low- density lipoprotein
OA: osteoarthritis
Acetyl-NF-κB p65 (Lys310): acetylation of Lysine 310
WT: wild Type
B6: C57BL/6
PBS: phosphate-buffered saline
micro-CT: micro-computed tomography
LysM: lysozyme M
α-MEM: serum-free minimum essential medium eagle alpha modifications
FBS: fetal bovine serum
ALP: Alkaline Phosphatase
ARS: alizarin red staining
siRNAs: small interfere RNAs
snRNA: small nucleolar RNA
β-gal: β-galactosidase
Tb. Th: trabecular thickness
VOI: volume of interest
Tb.Sp: trabecular separation
Tb.N: trabecular number
BV/TV: trabecular bone volume per tissue volume
Conn-Dens.: connectivity density
BFR: bone formation rate
MAR: mineral apposition rate
ELISA: enzyme-linked immunosorbent assay
N.Oc/B.Pm: numbers of osteoclasts
N.Ob/B.Pm: numbers of osteoblasts
MS/BS: mineralizing surface
AD: Alzheimer's disease
HUVECs: human umbilical vein endothelial cells
PI3K: PI3 kinase
VEGF: vascular endothelial growth factor
Tr.B: trabecular bone
