# Supplementary material for: miR-128 plays a critical role in murine osteoclastogenesis and estrogen deficiency-induced bone loss
Source: Theranostics. 2020 Mar 4;10(10):4334–48. doi: 10.7150/thno.42982 (PMC7150474; doi:10.7150/thno.42982)
Supplement: Supplementary file 1 — Supplementary figures and table. [file thnov10p4334s1.pdf]

Supplementary material

Supplementary Figure legends:

Supplementary Figure 1: Gene expression after overexpression, knockdown, or knockout. A, B The transfection efficiencies of miR-128 mimic or inhibitor. Data are mean ± SD. $^{**}P < 0.01$ by Student's *t* test. **C** *miR-128* levels in BMMs from *miR-128*$^{Oc-/-}$ mice. Data are mean ± SD. $^{**}P < 0.01$ by Student's *t* test. **D** Representative images of *WT* and *miR-128*$^{Oc-/-}$ male mice at 10 weeks old. **E** The efficiencies of siRNA of SIRT1. Data are mean ± SD. $^{**}P < 0.01$ by Student's *t* test.

Supplementary Figure 2: Cell proliferation and apoptosis assay. A BMMs were cultured in the presence of M-CSF and RANKL with miR-128 mimic or inhibitor. Data are mean ± SD. Not statistically significant by one-way ANOVA with Tukey's *post hoc* test. **B** BMMs were isolated from the *WT* and *miR-128*$^{Oc-/-}$ mice. Data are mean ± SD. Not statistically significant by Student's *t* test. **C** BMMs derived from *WT* and *miR-128*$^{Oc-/-}$ mice that were transfected with the siCtrl or siSIRT1. Data are mean ± SD. Not statistically significant by two-way ANOVA.

Supplementary Figure 3: Osteoclastic miR-128 deficiency does not affect osteoblastogenesis. A Representative H&E-stained sections (yellow arrows: osteoblasts) of trabecular bone (Tr.B) and histomorphometric analysis of osteoblast surfaces (Ob.S/BS). Scale bars: 100 μm. Data are mean ± SEM. Not statistically significant by Student's *t* test. **B** Mineralizing surface (MS/BS), mineral apposition rate (MAR), bone formation rate (BFR) were analyzed in calcein double-labeled plastic sections of mice vertebrae. Data are mean ± SEM. Not statistically significant by Student's *t* test. **C** Serum osteocalcin values tested by ELISA. Data are mean ± SEM. Not statistically significant by Student's *t* test. **D** Osteogenic differentiation of osteoblastic stromal cells harvested from *WT* and *miR-128*$^{Oc-/-}$ mice. ALP staining and ARS staining were carried out. **E-F** Quantitative analyses of the ALP activity and calcium mineralization. Data are mean ± SD. $^{**}P < 0.01$ by Student's *t* test. **G** qRT-PCR analysis of mRNA expression of *Runx2*, *Sp7*, *Alp*, and *Ocn*. Data are mean ± SD. $^{**}P < 0.01$ by Student's *t* test.

Supplementary Figure 4: Quantitative analyses of Western blotting results. A Osteoclastic miR-128 deficiency significantly increased SIRT1 protein level, while decreased p65-Acetyl 310 protein level. Data are mean ± SD. $^{**}P < 0.01$ by Student's *t* test. **B** SIRT1 knockdown in BMMs significantly recovered the decreased expression of p65-Acetyl 310 induced by osteoclastic miR-128 deficiency. Data are mean ± SD. $^{**}P < 0.01$ by two-way ANOVA.

Supplementary Figure 5: Osteoclastic miR-128 deficiency does not affect osteoblastogenesis in OVX mice. A Representative H&E-stained sections (yellow arrows: osteoblasts) of trabecular bone (Tr.B), and histomorphometric analysis of osteoblast surfaces (Ob.S/BS). Scale bars: 100 μm. Data are mean ± SEM. Not statistically significant by two-way ANOVA. **B** Mineralizing surface (MS/BS), mineral apposition rate (MAR), bone formation rate (BFR) were analyzed in calcein double-labeled plastic sections of mice vertebrae. Data are mean ± SEM. Not statistically significant by two-way ANOVA. **C** Serum osteocalcin values tested by ELISA. Data are mean ± SEM. Not statistically significant by two-way ANOVA.

Supplementary Figure 1:

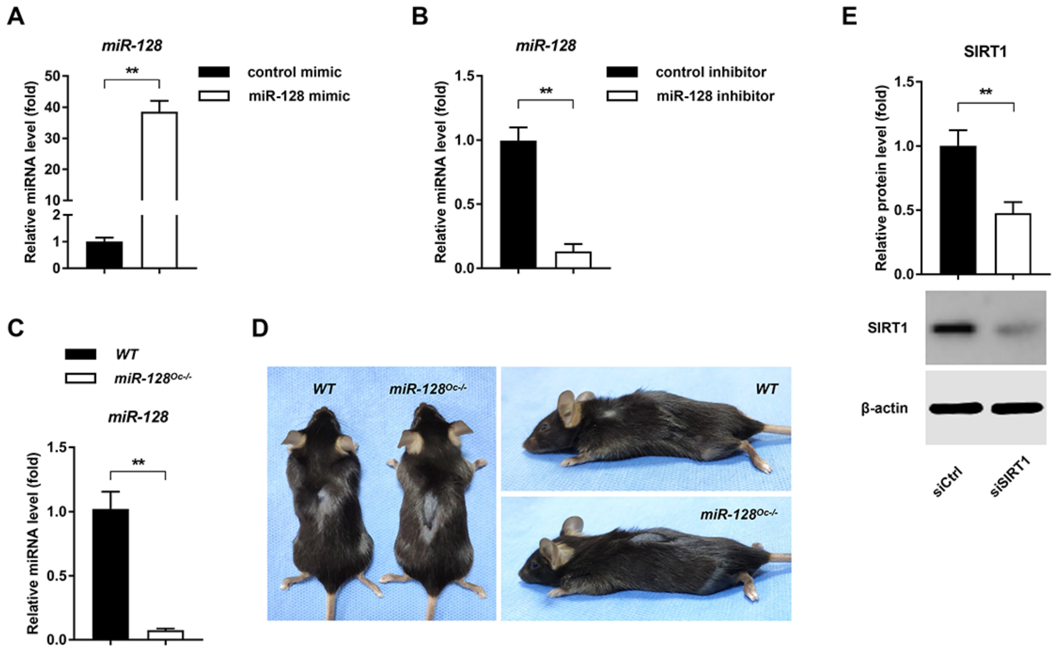

Supplementary Figure 2:

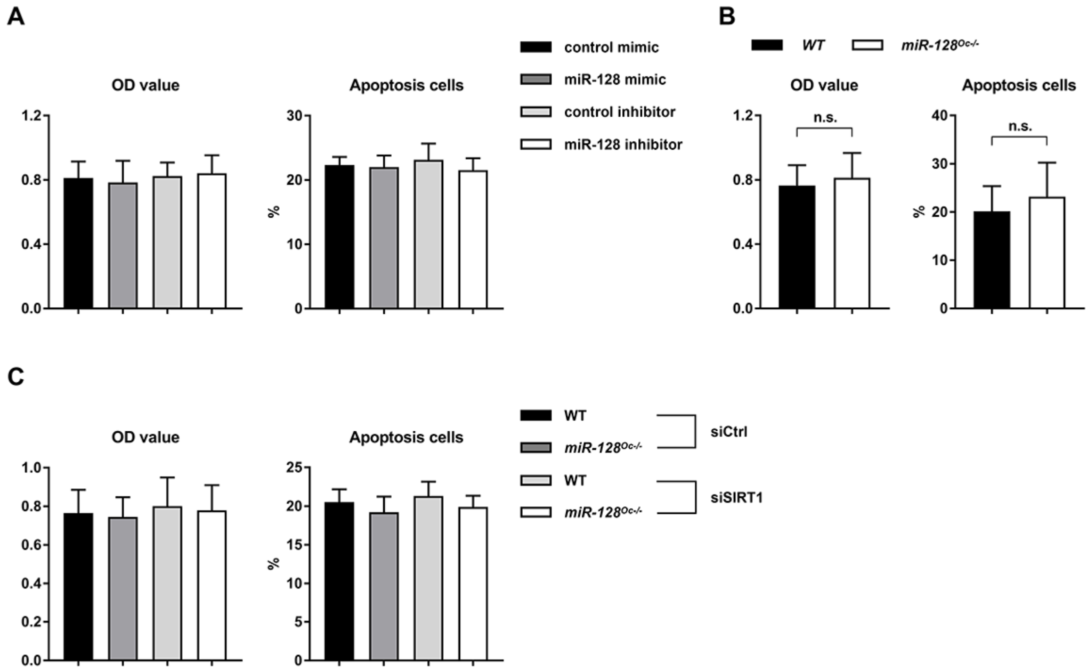

Supplementary Figure 3:

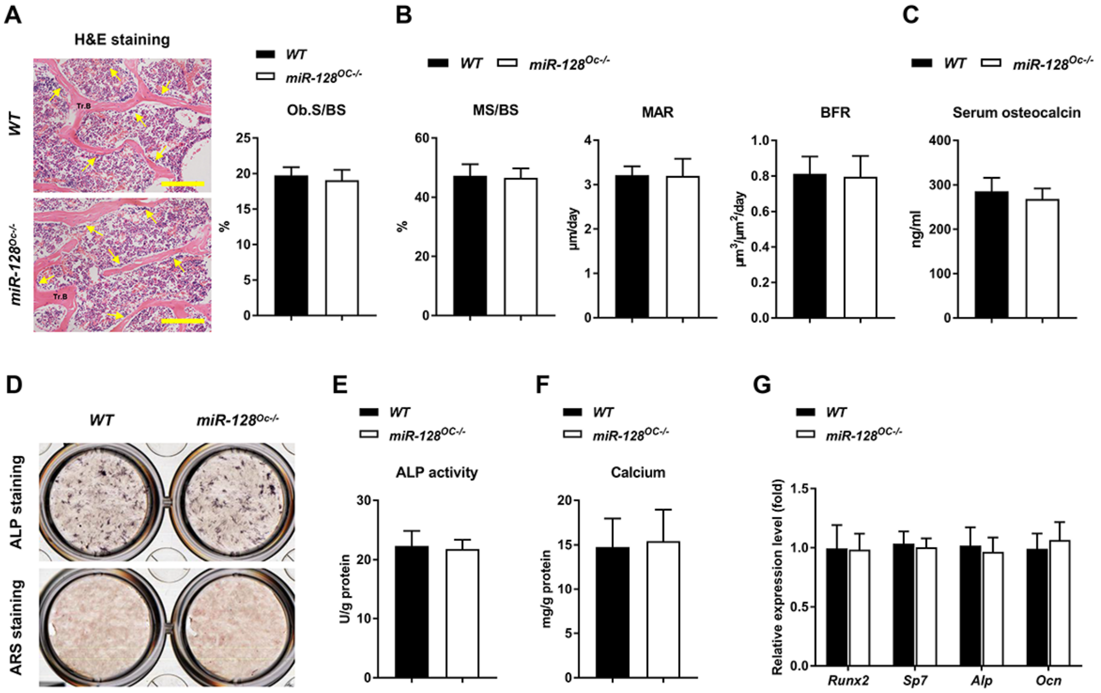

Supplementary Figure 4:

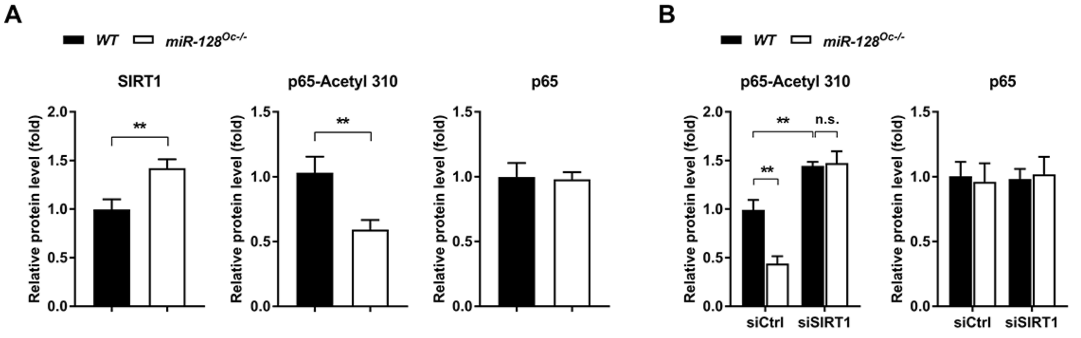

Supplementary Figure 5:

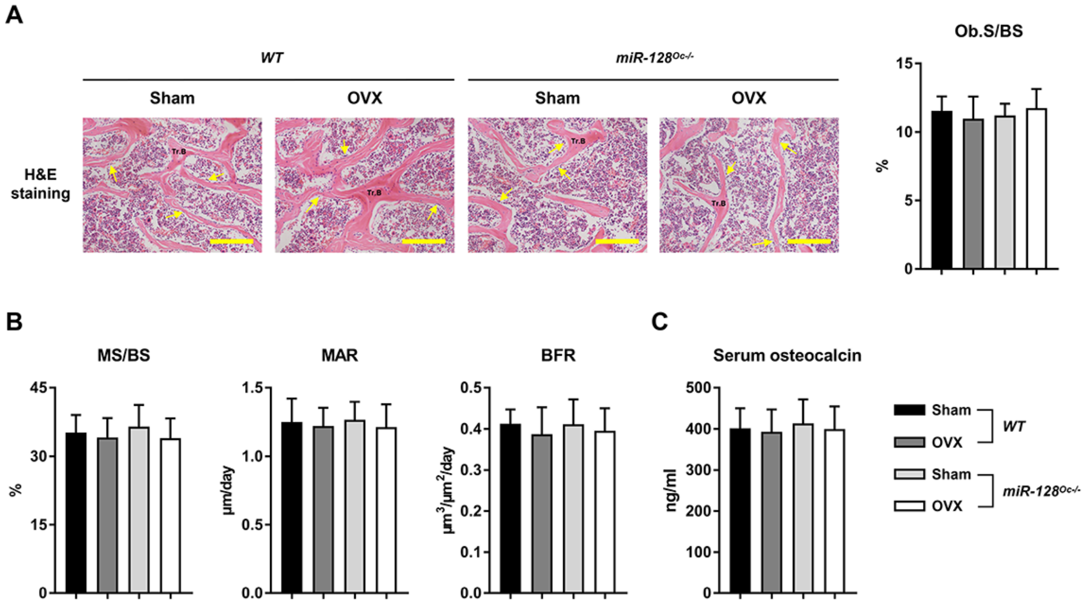

Supplementary Table 1. Sequences of siRNA, probe and primers.

Item	Sequence
siSIRT1	GCTGTTCGTGGAGACATTT
SIRT1 pull down probe	AAGGCGAGCATAGATACCGT
mmu-SIRT1 qRT-PCR primers	Forward: GCTGACGACTTCGACGACG
	Reverse: TCGGTCAACAGGAGGTTGTCT
mmu-miR-128 qRT-PCR primers	Forward: GGTCACAGTGAACCGGTC
	Reverse: GTGCAGGGTCCGAGGT
mmu-U6 qRT-PCR primers	Forward: GTGCTCGCTTCGGCAGCACATAT
	Reverse: AAAATATGGAACGCTTCACGAA
mmu-Nfatc1 qRT-PCR primers	Forward: CCCGTCACATTCTGGTCCAT
	Reverse: CAAGTAACCGTGTAGCTCCACAA
mmu-Traf6 qRT-PCR primers	Forward: AAAGCGAGAGATTCTTTCCCTG
	Reverse: ACTGGGGACAATTCACTAGAGC
mmu-Ctsk qRT-PCR primers	Forward: GAAGAAGACTCACCAGAAGCAG
	Reverse: TCCAGGTTATGGGCAGAGATT
mmu-c-Fos qRT-PCR primers	Forward: CGGGTTTCAACGCCGACTA
	Reverse: TTGGCACTAGAGACGGACAGA
mmu-c-Src qRT-PCR primers	Forward: GAACCCGAGAGGGACCTTC
	Reverse: GAGGCAGTAGGCACCTTTTGT
mmu-Tnf-α qRT-PCR primers	Forward: CCCTCACACTCAGATCATCTTCT
	Reverse: GCTACGACGTGGGCTACAG
mmu-IL-1 qRT-PCR primers	Forward: CGAAGACTACAGTTCTGCCATT
	Reverse: GACGTTTCAGAGGTTCTCAGAG
mmu-GAPDH qRT-PCR primers	Forward: ATCAAGAAGGTGGTGAAGCA
	Reverse: AGACAACCTGGTCCTCAGTGT
mmu-Runx2 qRT-PCR primers	Forward: GACCAGTCTTACCCCTCCTA
	Reverse: GGCAGTGTCATCATCTGAAA
mmu-Sp7 qRT-PCR primers	Forward: AAAGGAGGCACAAAGAAGC
	Reverse: CAGGAAATGAGTGAGGGAAG
mmu-Alp qRT-PCR primers	Forward: GCTTTAAACCCAGACACAAG
	Reverse: AAGAAGAAGCCTTTGAGGTT
mmu-Ocn qRT-PCR primers	Forward: CTCTCTCTGCTCACTCTGCT
	Reverse: GACTGAGGCTCCAAGGTAG
hsa-miR-128 qRT-PCR primers	Forward: ACACTCCAGCTGGGTCACAGTGAACCGGTC

	Reverse: TGGTGTCGTGGAGTCG
hsa-U6 qRT-PCR primers	Forward: CTCGCTTCGGCAGCACA
	Reverse: AACGCTTCACGAATTTGCGT
hsa-Nfatc1 qRT-PCR primers	Forward: AAAGACGCAGAAACGACG
	Reverse: TCTCACTAACGGGACATCAC
hsa-GAPDH qRT-PCR primers	Forward: ATCAAGAAGGTGGTGAAGCA
	Reverse: GTCGCTGTTGAAGTCAGAGGA
